# Prevention of Cerebral Lesion in Asymptomatic Carotid and Vertebral Artery Stenosis in Urgent Cardiac Surgery

**DOI:** 10.21470/1678-9741-2020-0445

**Published:** 2021

**Authors:** Ignazio Condello, Salvatore Condello

**Affiliations:** 1 Department of Cardiac Surgery, Anthea Hospital, GVM Care & Research, Bari, Italy. E-mail: ignicondello@hotmail.it; 2 Neuromotor Rehabilitation Unit; Istituti Clinici Scientifici Maugeri Spa, Ribera (AG), Italy.

Carotid artery stenosis (CAS) and vertebral artery stenosis (VAS) are associated with cerebral infarction after cardiopulmonary bypass (CPB). We read with great interest the article “Cerebral Lesions in Patients Undergoing Coronary Artery Bypass Grafting in Relation to Asymptomatic Carotid and Vertebral Artery Stenosis”, by Wiberg et al. ^[1]^ The authors present a study that included patients treated for CABG procedures, in which CAS and VAS were identified by magnetic resonance angiography. Cerebral magnetic resonance imaging was performed to identify new post-operative subclinical cerebral lesions. Associations between CAS/VAS post-operative cerebral lesions were investigated. Forty-six patients were included in the study. Thirteen percent had significant CAS and 11% had significant VAS. Thirty-five percent had new cerebral infarction postoperatively. The authors found a significant association between the presence of cerebral vessel stenosis and acute cerebral infarction. In this letter to the editor, we emphasize that intraoperative management and monitoring during CPB could be crucial, in particular for patients with CAS and VAS who need urgent cardiac surgery. The optimal management of such patients remains controversial. Gold et al. ^[2]^ showed that high mean arterial pressure (MAP) during CPB reduces the overall incidence of combined cardiac and neurological complications. Johnsson et al. ^[3]^ did not show an increased incidence of perioperative neurologic events with a MAP >70 mmHg during CPB in patients with significant bilateral CAS. Maintaining cerebral regional oxygen saturation index (rSO2) in preoperative range reduces the incidence of postoperative cognitive dysfunction and general morbidity. Near-infrared spectroscopy (NIRS) allows assessment of cerebral oxygen delivery and the demands ratio to the frontal cortex. Routine use of cerebral rSO2 monitoring during cardiac surgery may improve patient’s outcome and shorten hospital stay. Cerebral monitoring using NIRS can identify otherwise unrecognized cerebral hypoperfusion during aortic surgery as well. NIRS has been extensively used in patients undergoing cardiac surgery to find an association between the measurements of cerebral oxygenation and postoperative outcome ^[4]^. Several studies have found an association between intraoperative cerebral oxygen desaturation and postoperative cognitive dysfunction, stroke, and prolonged hospital stay ^[5]^. There is no evidence base for the management of patients with asymptomatic CAS and VAS requiring urgent cardiac surgery. Only a large multicenter international randomized study can provide an evidence base for the management of these patients.
